# Diversity, Bacterial Symbionts, and Antimicrobial Potential of Termite-Associated Fungi

**DOI:** 10.3389/fmicb.2020.00300

**Published:** 2020-03-13

**Authors:** Xiao Xu, Mingwei Shao, Caiping Yin, Zhenchuan Mao, Jingjing Shi, Xinyuan Yu, Ying Wang, Feifei Sun, Yinglao Zhang

**Affiliations:** ^1^School of Life Sciences, Anhui Agricultural University, Hefei, China; ^2^College of Chemistry and Life Science, Zhejiang Normal University, Jinhua, China; ^3^Institute of Vegetables and Flowers, Chinese Academy of Agricultural Sciences, Beijing, China

**Keywords:** *Odontotermes formosanus*, fungal diversity, bacterial symbionts, antimicrobial activities, secondary metabolites

## Abstract

The phylogenetic diversity of fungi isolated from the *Odontotermes formosanus* was investigated by dilution-plate method, combined with morphological characteristics and 5.8S rDNA sequencing. Thirty-nine fungi were isolated and purified from *O. formosanus*, which were belonging to two phyla and four classes (Sordariomycetes, Dothideomycetes, Eurotiomycetes, Agaricomycetes). Furthermore, nine bacterial 16S rRNA sequences were obtained from total fungal genomic DNA. All bacterial symbionts were segmented into four genera: *Bacillus*, *Methylobacterium*, *Paenibacillus*, and *Trabulsiella.* The antimicrobial activities of all endophytic fungi extracts were tested by using the filter paper method against *Escherichia coli* (ATCC 8739), *Bacillus subtilis* (ATCC 6633), *Staphylococcus aureus* (ATCC 6538), and *Canidia albicans* (ATCC 10231). The results exhibited that 25 extracts (64%) exhibited antibacterial activity against at least one of the tested bacterial strains. Furthermore, the secondary metabolites **1** [5-hydroxyramulosin **(1a)**:biatriosporin M **(1b)** = 2:1] from the *Pleosporales* sp. BYCDW4 exhibited potent antimicrobial activities against *E. coli*, *C. albicans*, *B. subtilis*, and *S. aureus* with the inhibition zone diameter (IZD) of 13.67, 14.33, 12.17, and 11.33 mm, respectively, which were comparable with those of the positive control. 1-(2,5-Dihydroxyphenyl)-3-hydroxybutan-1-one (**2**) from the *Microdiplodia* sp. BYCDW8 showed medium inhibitory activities against *B. subtilis* and *S. aureus*, with the IZD range of 8.32–9.13 mm. In conclusion, the study showed the diversity of insect symbionts could be expected to develop the resource of new species and antibiotics.

## Introduction

The symbiotic relationships, kinds of organisms including microorganisms, animals, and plants can be seen everywhere in the world (Blackwell and Vega, 2018; Grigorescu et al., 2018). For example, the first direct visual evidence of a not-yet-cultured CFB bacterium is detected in a mycorrhizal fungus (Barbieri et al., 2000). Many others with well-studied examples including the sponge and symbiotic strains, nitrogen-fixing bacteria, and legumes live in symbiotic association (Trainer and Charles, 2006; Webster et al., 2008). Compared with other interactions, the insect symbiont interactions still represent a largely unknown field (Park et al., 2018).

There are few researches reported that symbioses of insects are an important unexploited microbial resource for human beings. For example, insect gut microbes, especially fungi in suppression of unwanted microorganisms (parasites, microorganic pathogens, etc.), protecting the hosts and maintaining the ordinary growth and behavior, are of interest to insects (Lu et al., 2016; Beemelmanns et al., 2017; Van Arnam et al., 2017). Based on this symbiosis, several new immunosuppressive polyketides were obtained from a fungus attached to mantis, *Daldinia eschscholzii* IFB-TL01 (Zhang et al., 2008, 2011). In addition, insect symbiosis is also an important source of new microorganisms. For instance, 57 entomopathogenic fungi were analyzed, including one species which was firstly reported (Nishi and Sato, 2017). Many new species were also isolated from aquatic insects, among which new genera were found (White and Lichtwardt, 2004; White et al., 2018). Nonetheless, extensive, methodical, and biologically correlative researches about the insect microorganisms were restricted, especially when the complexity of insect symbiotic system was taken into consideration.

Termites, belonging to the class Insecta and the order Isoptera in the phylum Arthropoda, are a kind of eusocial and hemimetabolous insects (Mathew et al., 2012). Multitudinous and particular microbial floras reside in the termites, most of which can help the host to digest and utilize their food (Zhu et al., 2012; Li et al., 2017). *Odontotermes formosanus* as a termite species is mainly distributed in south areas of the line between Yellow River and Yangtze River in China. As far as we know, research concentrated on *O. formosanus* fungi derived from China have been rather rare (Chou et al., 2007; Shinzato et al., 2007). Underexploited *O. formosanus* symbionts can offer a pathway to finding unreported biological resources, which will solve the dilemma that needs to be dealt with regarding new drugs, especially antibiotics, which we are in sore need of due to increasing bacterial resistance to existing antibiotics (Procópio et al., 2012; Bi et al., 2013; Elahwany et al., 2013). Herein, the study was intended to explore the phylogenetic diversity, bacterial symbionts, antimicrobial potency, and compounds of the fungi associated with *O. formosanus*.

## Materials and Methods

### Sample Collection and Microbial Isolations

A total of 50 termite samples were collected from the rotten wood in a grove in Zhejiang Normal University (29°00′17.37^″^N, 119°29′54.84^″^E, Jinhua city, China) during early spring in March 2016. The 50 collected samples were starved for 12 h and surface-sterilized in 75% ethanol for 2 min, followed by rinsing in sterilized water three times (30 s each). Sterile forceps were used to dissect samples to get the guts. The guts and degutted bodies of termites were fully homogenized separately in 0.5 ml sterile water. Then, the homogenates were diluted in a 10-fold series (i.e., 10^–1^, 10^–2^, 10^–3^), and aliquots of 200 μl from each dilution were spread onto 10 isolation media (Supplementary Table S1). Pure colonies of fungi from the appropriate dilution were transferred into new MEA medium. Isolated strains were preserved on MEA slants at 4°C until use. Fungal species were grouped via molecular sequence data. The fungi were stored at our institute.

### DNA Sequencing

DNA sequencing was performed according to the methods detailed previously (Hoffman and Arnold, 2010; Shao et al., 2015). Symbiotic fungi were transferred into ME medium (20 g malt extract, 20 g sucrose, 1 g peptone in 1 L of distilled H_2_O) and cultured at 28 ± 0.5°C on rotary shakers for 5–6 days. The growing fungal mycelia were used to afford samples for genomic DNA extraction. Fungal total DNA was amplified using the Fast DNA Extraction Kit (BioTeke, Beijing, China) as claimed by the manufacturer’s specification. The genomic DNA was stored at 4°C until use. The primers ITS1/ITS4 and 27F/1492R were used to amplify 5.8S rDNA and 16S rRNA based on the fungal genomic DNA. Qualified PCR samples were used for sequencing (Sangon Biotech Co., Ltd., Shanghai, China).

### Fungi’s Identification and Phylogenetic Analyses

As described previously (Shao et al., 2015), all resulting sequences’ affiliation which were returned from Sangon Biotech Company were recognized by the available data in BLAST from the National Center for Biotechnology Information (NCBI) database. Sequence alignment and neighbor-joining phylogenetic analysis were carried out using MEGA software version 5.1. Bootstrap analysis of tree construction on the strength of the sequences was accustomed to estimate the neighbor-joining information based on 1,000 replicates (Felsenstein, 1985). The 5.8S rDNA sequences that had been obtained were placed in NCBI with the accession numbers MG820065–MG820103. Besides, endosymbiotic bacterial 16S rRNA sequences were deposited in NCBI with the accession numbers MG825089–MG825096.

### Fermentation

Every fungus was grown on MEA medium at 28 ± 0.5°C for 3–4 days. Then, pieces of fresh mycelium were inoculated into 1-L Erlenmeyer flasks each containing 150 ml of ME liquid medium, after 2–3 days of incubation at 28 ± 0.5°C in a shaker rotating at 180 rpm. A 20-ml suspension of the fungus was transferred as seed into 1-L Erlenmeyer flasks each containing 400 ml of ME liquid medium. The flask cultures were incubated at 28 ± 0.5°C for 1 week.

### Isolation of Compounds From BYCDW4 and BYCDW8

A total of 26 L of fermentation broth of BYCDW4 was filtered and extracted with EtOAc (3 × 26 L) at room temperature. The solvent was then evaporated *in vacuo* to give a black-brown crude extract (13.5 g). The obtained extract was separated by column chromatography (CC) using silica gel (SiO_2_: 200–300 mesh, 15 g) and eluting with a stepwise gradient of CH_2_Cl_2_/MeOH (100:0–100:16, *v*/*v*) to afford six primary fractions (A to F). Then the white needle crystal (compound **1**, 5.84 g) were recrystallized by CH_2_Cl_2_/MeOH continuously up to no impurity.

The dark-yellow mixture of BYCDW8 (1.5 g) was obtained with the method as above. Compound **2** (14.5 mg) was isolated and purified from subfraction B-2, which was given from further chromatography over silica gel in fraction B (0.4 g, CH_2_Cl_2_/MeOH, 100:1).

### Structural Elucidation of Metabolites

The structures of compounds **1** and **2** were primarily analyzed by mass and ^1^H/^13^C-nuclear magnetic resonance (NMR) spectroscopies. The electrospray ionization mass spectrometry of the purified metabolites was recorded on a TripeTOF 4600 instrument (Bruker, Billerica, MA, United States). ^1^H/^13^C NMR spectra were measured with a Bruker AVANCE-600 (Bruker, Switzerland) spectrometer, and chemical shifts were reported as parts per million (δ) by referring to tetramethylsilane as internal standards. The structure of compound **1a** was further determined by single crystal X-ray.

### Antimicrobial Activities

The antimicrobial activities of all the 39 fungal crude extracts and the secondary metabolites were evaluated using filter-paper method as described previously (Li et al., 2014). Three tested pathogens (*Escherichia* coli ATCC 8739, *Bacillus subtilis* ATCC 6633, *Staphylococcus aureus* ATCC 6538) were cultivated on NA medium (NaCl 3 g, peptone 10 g, beef extract 3 g, agar 18–20 g, distilled H_2_O 1,000 ml) in 37°C, whereas the pathogenic *Candida albicans* (ATCC 10231) was inoculated on PDA medium in 28°C. Then 200 μl of the overnight fermentation broth of the pathogens was inoculated on the corresponding plate. Next, 5 μl of the tested objects including crude extracts, metabolites, and positive control drugs which were dissolved completely by acetone with the concentration of 6 mg/ml was pipetted onto a sterile filter disk with a diameter of 5 mm. Gentamicin sulfate and amphotericin B were used as the positive control of pathogenic bacteria and yeast, respectively. Then, all the processed filter papers were placed on the above pre-prepared medium. Each test was set up three repeats. Plates were cultivated at constant temperature incubator and the inhibition zone was measured at 24–36 h.

## Results

### Phylogenetic Diversity of Cultivable Fungi From *O. formosanus*

**FIGURE 1:**
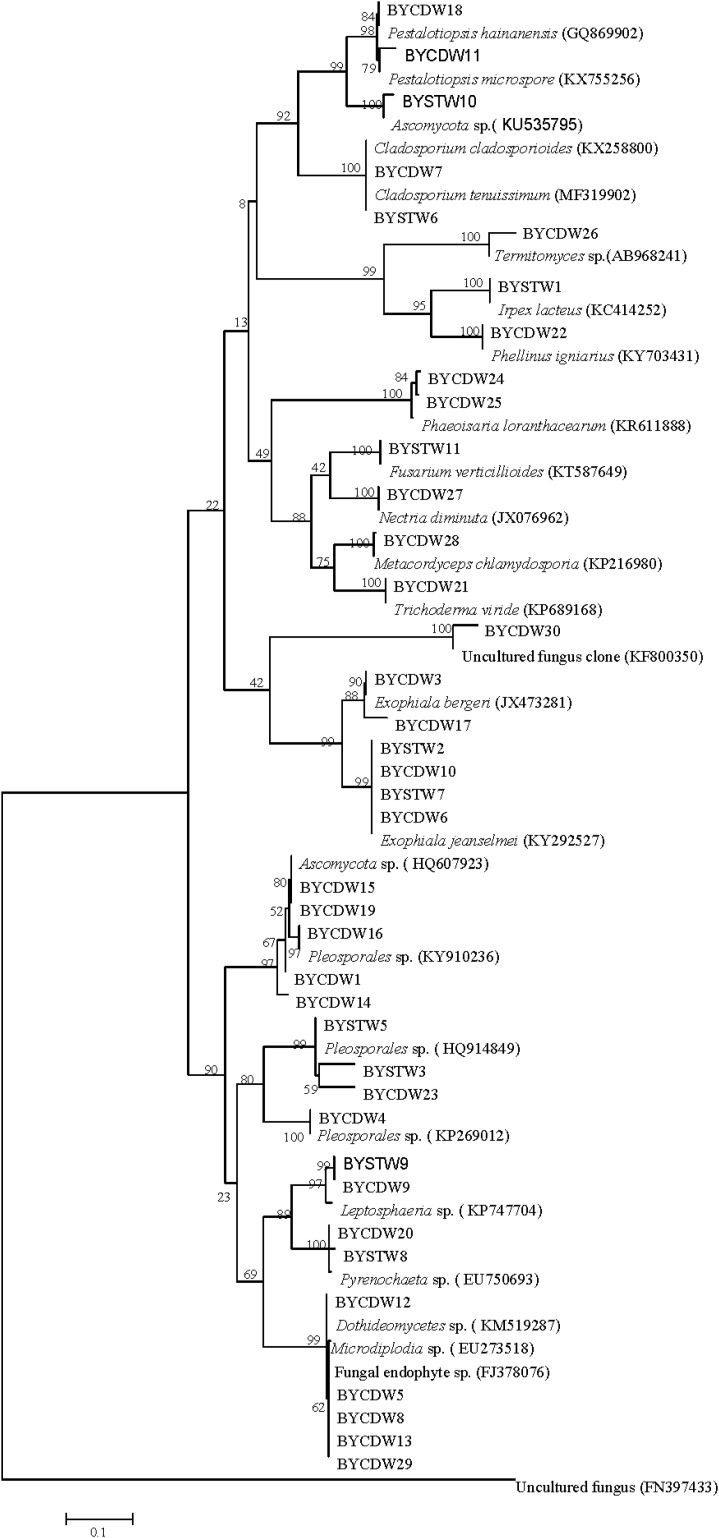
Neighbor-joining phylogenetic tree of ITS rDNA sequences of *O. formosanus* fungal isolates. Bootstrap values were calculated using 1,000 replications.

In this study, a total of 39 symbiotic fungi were isolated from *O. formosanus* (Table 1 and Figure 1). The ITS1-5.8S-ITS2 region of all strains were sequenced and compared with available GenBank reference sequences. Sequences analysis showed that all fungi were attached to the phyla Ascomycota and Basidiomycota, 35 strains of which were grouped into three classes [Sordariomycetes (23.1%), Dothideomycetes (53.8%), and Eurotiomycetes (15.4%)] within Ascomycota. The other three strains (8%) were distributed in the Agaricomycetes within the phylum Basidiomycota (Figure 1).

**TABLE 1:** Phylogenetic analysis of cultivable fungi associated with *O. formosanus.*

Isolate code	Closest match	Accession no.	Proposed identity	Coverage/max ident	GenBank no.
BYCDW15	*Ascomycota* sp.	HQ607923	*Ascomycota* sp.	97/99	MG820065
BYCDW19	*Ascomycota* sp.	HQ607923	*Ascomycota* sp.	99/99	MG820066
* BYCDW1	*Ascomycota* sp.	HQ607923	*Ascomycota* sp.	99/97	MG820067
BYSTW10	*Ascomycota* sp.	KU535795	*Ascomycota* sp.	97/98	MG820068
BYCDW7	*Cladosporium cladosporioides*	KX258800	*Cladosporium cladosporioides*	100/99	MG820069
BYSTW6	*Cladosporium cladosporioides*	MF319902	*Cladosporium cladosporioides*	99/99	MG820070
BYCDW12	*Dothideomycetes* sp.	KM519287	*Dothideomycetes* sp.	98/99	MG820073
BYCDW3	*Exophiala bergeri*	JX473281	*Exophiala bergeri*	94/99	MG820091
* BYCDW17	*Exophiala bergeri*	JX473281	*Exophiala bergeri*	95/94	MG820075
BYSTW2	*Exophiala jeanselmei*	KY292527	*Exophiala jeanselmei*	98/99	MG820076
BYCDW10	*Exophiala jeanselmei*	KY292527	*Exophiala jeanselmei*	98/99	MG820077
BYSTW7	*Exophiala jeanselmei*	KY292527	*Exophiala jeanselmei*	98/99	MG820078
BYCDW6	*Exophiala jeanselmei*	KY292527	*Exophiala jeanselmei*	96/99	MG820079
BYCDW13	*Paraconiothyrium brasiliense*	FJ378076	*Paraconiothyrium brasiliense*	97/99	MG820080
BYCDW29	*Paraconiothyrium brasiliense*	FJ378076	*Paraconiothyrium brasiliense*	98/99	MG820081
BYSTW11	*Fusarium verticillioides*	KT587649	*Fusarium verticillioides*	99/99	MG820082
BYSTW1	*Irpex lacteus*	KC414252	*Irpex lacteus*	98/99	MG820083
* BYSTW9	*Leptosphaeria* sp.	KP747704	*Leptosphaeria* sp.	99/96	MG820084
* BYCDW9	*Leptosphaeria* sp.	KP747704	*Leptosphaeria* sp.	98/96	MG820085
BYCDW28	*Metacordyceps chlamydosporia*	KP216980	*Metacordyceps chlamydosporia*	96/99	MG820086
BYCDW5	*Microdiplodia* sp.	EU273518	*Microdiplodia* sp.	98/99	MG820071
BYCDW8	*Microdiplodia* sp.	EU273518	*Microdiplodia* sp.	98/99	MG820072
BYCDW27	*Nectria diminuta*	JX076962	*Nectria diminuta*	88/99	MG820087
* BYCDW30	*Ochroconis constricta*	KM056323	*Ochroconis constricta*	97/92	MG820088
* BYSTW5	*Pleosporales* sp.	HQ914849	*Pleosporales* sp.	97/99	MG820089
* BYCDW4	*Pleosporales* sp.	KP269012	*Pleosporales* sp.	99/99	MG820092
* BYSTW3	*Pleosporales* sp.	HQ914849	*Pleosporales* sp.	98/93	MG820090
* BYCDW20	*Pyrenochaeta* sp.	KJ207418	*Pyrenochaeta* sp.	97/99	MG820093
BYSTW8	*Pyrenochaeta* sp.	EU750693	*Pyrenochaeta* sp.	97/99	MG820094
BYCDW16	*Pleosporales* sp.	KY910236	*Pleosporales* sp.	97/100	MG820095
* BYCDW23	*Pleosporales* sp.	HQ914849	*Pleosporales* sp.	80/94	MG820091
BYCDW18	*Pestalotiopsis hainanensis*	GQ869902	*Pestalotiopsis hainanensis*	99/99	MG820096
* BYCDW24	*Phaeoisaria loranthacearum*	KR611888	*Phaeoisaria loranthacearum*	99/96	MG820097
* BYCDW25	*Phaeoisaria loranthacearum*	KR611888	*Phaeoisaria loranthacearum*	97/97	MG820098
BYCDW11	*Pestalotiopsis microspora*	KX755256	*Pestalotiopsis microspora*	97/97	MG820099
BYCDW22	*Phellinus igniarius*	KY703431	*Phellinus igniarius*	98/99	MG820100
BYCDW21	*Trichoderma viride*	KP689168	*Trichoderma viride*	98/100	MG820102
* BYCDW26	*Termitomyces* sp.	AB968241	*Termitomyces* sp.	98/95	MG820101
* BYCDW14	Uncultured fungus	FN397433	*Ascomycota* sp.	98/98	MG820103

* Potential new strain.

The fungi of Dothideomycetes were dominant species in phylogenetic diversity of cultivable fungi from *O. formosanus*. The largest number (21) of isolates was distributed in three orders, including the Capnodiales, Venturiales, and Pleosporales. Among of them, two strains belonging to Capnodiales showed highly similar to *Cladosporium cladosporioides* with identity of 99%. The strain BYCDW30 showed only 92% similarity to *O. constricta*, which indicated a potential new species. The most frequent order identified was the Pleosporales, which included six genera based on phylogenetic tree analysis (Figure 1).

The strains of Sordariomycetes (9) were mainly included in the three classes (Xylariales, Pleurotheciales, and Hypocreales). Four of them were grouped into the family Hyporcreaceae and were identified as *Fusarium verticillioides*, *Metacordyceps chlamydosporia*, *Nectria diminuta*, and *Trichoderma viride*, respectively (Figure 1). Two strains showed similar to *Phaeoisaria loranthacearum* with a low identity of 96%. Two other strains belonging to Xylariales were identified as *Pestalotiopsis hainanensis* and *P. microspora*, respectively.

Six isolates of the class Eurotiomycetes were mainly concentrated in the genus *Exophiala* grouped into the order Chaetothyriales. Four strains showed highly similar to *Exophiala jeanselmei* with an identity of more than 99%. Another two strains were similar to *E. bergeri* with sequence match of 94% and 99%, respectively. What was worth mentioning was that the BYCDW17 showed a sequence match of 94% with *E. bergeri*, which indicated that it might not have been deposited in the GenBank database or represent a potential new species (Landeweert et al., 2003).

The fungi of class Agaricomycetes belonging to the phylum Basidiomycota were mainly distributed in the orders Agaricales and Polyporales. The strain BYCDW26 of Agaricales was a *Termitomyces* fungus, which had the low similar sequence with an identity of 88.10%. Another two strains belonging to Polyporales were identified as *Irpex lacteus* and *Phellinus igniarius*, respectively.

### Diversity of Bacterial Symbionts

A total of nine bacterial symbionts 16S rRNA was obtained from the fungi associated with *O. formosanus* in this study (Supplementary Table S2). These bacterial symbionts mainly pertained to the class Dothideomycetes and identified as Bacillales (*Bacillus*, *Paenibacillus*), Rhizobiales (*Methylobacterium*), and Enterobacterium (*Trabulsiella*). Furthermore, six bacteria were putatively divided into genus *Bacillus*.

### Antimicrobial Activities of the Crude Extracts of Fungi

The antimicrobial results are shown in Table 2. Of the all isolates, 25 extracts (64%) exhibited antibacterial activity against at least one of the tested bacterial strains. Especially, BYCDW8, BYCDW4 (Supplementary Figure S1), BYCDW13, and BYCDW19 exhibited remarkable inhibitory activities against *S. aureus* (ATCC 6538) with the inhibition zone diameter (IZD) of more than 20 mm. These four fungi were all attached to Pleosporales in the class Dothideomycetes. Furthermore, the strain BYCDW4 possessed notable inhibitory effect against *E. coli* (ATCC 8739) with the IZD of 21.07 mm, which was stronger than the positive gentamicin sulfate with the IZD of 17.67 mm. However, other strains did not express remarkable activity against *E. coli.* The crude extracts of BYCDW3, BYCDW4, and BYCDW25 exhibited great inhibition effect on *C. albicans* (ATCC 10231), which were equivalent to that of amphotericin with the IZD of 19.17 mm. And among them, the crude extract of BYCDW25 was the most prominent. Further analysis showed that the inhibition activities of the fungal extracts against gram-positive bacteria was much better than the gram-negative bacteria. In addition, the potential new isolates BYCDW24 and BYCDW25 (in the order Pleurotheciales) presented moderate antimicrobial activities against several pathogens.

**TABLE 2:** Antimicrobial activities of 39 fungal crude extracts against pathogenic bacteria and yeast (mm).

Strains	*E. coli*	*C. albicans*	*B. subtilis*	*S. aureus*
BYCDW11	NI	NI	NI	7.83 ± 0.29
BYCDW3	8.17 ± 1.53	15.83 ± 1.15	11.50 ± 1.00	14.33 ± 2.57
BYCDW6	NI	NI	12.47 ± 0.38	14.90 ± 0.26
BYCDW23	14.00 ± 1.50	12.30 ± 2.25	12.13 ± 1.18	13.20 ± 1.25
BYCDW24	12.83 ± 1.26	NI	15.28 ± 6.26	NI
BYCDW5	11.67 ± 0.58	NI	14.75 ± 0.37	11.33 ± 1.53
BYCDW8	6.53 ± 0.25	NI	14.00 ± 1.80	22.33 ± 3.21
BYCDW4	21.07 ± 0.26	14.80 ± 0.84	18.45 ± 1.04	21.38 ± 0.12
BYCDW1	NI	NI	NI	8.03 ± 0.40
BYCDW29	11.23 ± 1.25	NI	NI	7.33 ± 0.21
BYCDW13	NI	NI	17.30 ± 0.83	20.4 ± 0.61
BYCDW25	NI	17.58 ± 0.60	11.55 ± 0.05	14.85 ± 0.26
BYCDW14	NI	NI	NI	NI
BYCDW20	NI	NI	NI	NI
BYCDW18	NI	NI	NI	NI
BYCDW19	NI	NI	NI	20.27 ± 0.64
BYCDW10	10.97 ± 0.15	NI	10.87 ± 0.15	NI
BYCDW26	NI	NI	NI	NI
BYCDW16	NI	NI	NI	NI
BYCDW30	NI	NI	NI	7.03 ± 0.12
BYCDW21	NI	NI	NI	6.97 ± 0.06
BYCDW28	NI	NI	NI	NI
BYCDW7	NI	NI	NI	NI
BYCDW9	NI	NI	NI	7.73 ± 0.25
BYCDW22	7.73 ± 0.06	NI	NI	NI
BYCDW15	NI	NI	NI	NI
BYCDW17	NI	NI	NI	NI
BYCDW12	NI	NI	NI	7.47 ± 0.46
BYCDW27	NI	10.02 ± 0.14	7.11 ± 0.14	12.42 ± 0.50
BYSTW11	12.67 ± 1.76	12.33 ± 0.76	12.33 ± 0.76	12.83 ± 0.76
BYSTW6	7.13 ± 0.85	NI	NI	6.40 ± 0.26
BYSTW1	NI	NI	10.68 ± 0.51	14.12 ± 0.83
BYSTW9	NI	NI	6.95 ± 0.10	7.06 ± 0.10
BYSTW7	NI	NI	6.67 ± 0.19	6.65 ± 0.10
BYSTW8	NI	NI	NI	NI
BYSTW2	NI	NI	NI	NI
BYSTW10	NI	NI	NI	NI
BYSTW3	8.40 ± 1.70	NI	NI	9.20 ± 1.84
BYSTW5	NI	NI	NI	10.53 ± 0.80
PC^a^	17.67 ± 0.29	19.17 ± 0.76	19.17 ± 0.29	21.33 ± 0.58

*^*a*^PC as the positive control, gentamicin sulfate and amphotericin as the positive control of pathogenic bacteria and yeast, respectively; results are presented as the mean ± standard; “NI” means not inhibited; the concentration for the test is 30 μg/filter paper.*

### Identification of the Secondary Metabolites Isolated From BYCDW4 and BYCDW8

**FIGURE 2:**
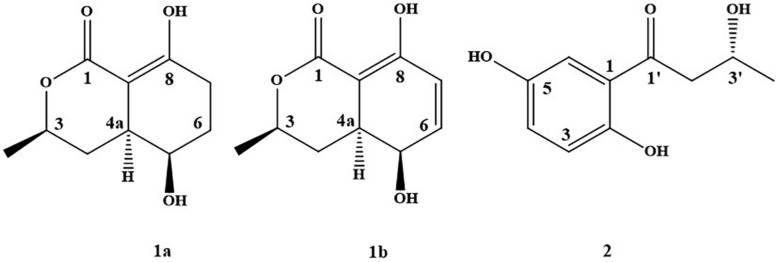
The structure of compounds **1** and **2**.

Secondary metabolites were identified by spectroscopic analyses, including HR–ESI-MS, NMR, and comparisons with the data described in the previous literatures. The constituents were identified as compound **1** [5-hydroxyramulosin **(1a)**: biatriosporin M **(1b)** = 2:1] from BYCDW4 and 1-(2,5-dihydroxyphenyl)-3-hydroxybutan-1-one (**2**) (Figure 2) from BYCDW8 based on the following data.

Compound **1** was obtained as a white needle crystal, which is a mixture (5-hydroxyramulosin (Osterhage et al., 2002) and biatriosporin M (Zhou et al., 2016)) with the proportion of 2:1 from the data of ^1^H NMR. 5-Hydroxyramulosin (**1a**): HR–ESI-MS: [M + H]^+^ *m/z* 199.0964 (calcd for 199.0970), [M + Na]^+^ *m/z* 221.0785 (calcd for 221.0790); ^1^H NMR (CDCl_3_) δ: 13.30 (s, 1H), 4.48 (m, 1H), 4.05 (m, 1H), 2.68 (m, 1H), 2.59 (m, 1H), 2.38 (m, 1H), 2.05 (m, 1H), 1.72 (m, 1H), 1.96 (m, 1H), 1.84 (m, 1H), 1.42 (d, 6.3, 3H); ^13^C NMR (CDCl_3_) δ: 174.3, 172.4, 92.2, 76.2, 66.0, 37.4, 32.7, 27.9, 24.3, 21.8. Biatriosporin M (**1b**): HR–ESI-MS: [M + H]^+^ *m/z* 197.0807 (calcd for 197.0814), [M + Na]^+^ *m/z* 219.0625 (calcd for 219.0633); ^1^H NMR (CDCl_3_) δ: 12.70 (s, 1H), 6.66 (dd, 9.9, 5.4, 1H), 6.25 (d, 9.9, 1H), 4.50 (m, 1H), 3.99 (m, 1H), 2.81 (m, 1H), 1.88 (m, 1H), 1.80 (m, 1H), 1.47 (d, 6.3, 3H); ^13^C NMR (CDCl_3_) δ: 172.5, 166.0, 138.7, 125.8, 90.9, 75.9, 63.2, 36.0, 30.3, 21.6. Compound **1a** was further identified based on single-crystal X-ray diffraction data (Supplementary Table S3 and Supplementary Figure S2). Compound **1b** was a dehydrogenated derivative of **1a**. Owing to their similar polarity, we were not able to isolate them by conventional separation methods. To the best of our knowledge, this was the first report that compound **1** was isolated from the fungus with high yield of 43.3% (compound/crude extract = 5.84 g:13.50 g). Worth mentioning was that compound **1a** (5-hydroxyramulosin) was reported with good antifungal activity against the fungal pathogen *Aspergillus niger* (IC_50_ = 2.10 μg/ml) and remarkable cytotoxicity to murine leukemia cells (IC_50_ = 2.10 μg/ml) (Santiago et al., 2012).

1-(2,5-Dihydroxyphenyl)-3-hydroxybutan-1-one (**2**) (Hong et al., 2018): light green-orange powder. HR-ESI-MS: [M + H]^+^ *m/z* 197.0804 (calcd for 197.0814).^1^H NMR (acetone-*d*6) δ: 11.77 (br s, 1H), 8.19 (br s, 1H), 7.34 (d, 2.94, 1H), 7.09 (dd, 2.94, 8.88, 1H), 6.80 (d, 8.88, 1H), 4.37 (m, 1H), 3.92 (br s, 1H), 3.19 (dd, 8.04, 17.52, 1H), 3.03 (dd, 4.62, 17.52, 1H), 1.26 (d, 6.20, 3H). ^13^C NMR (acetone-*d*6) δ: 206.6, 156.6, 150.4, 125.6, 120.3, 119.3, 115.9, 64.8, 48.2, 23.7.

### Antimicrobial Activities of Secondary Metabolites Isolated From BYCDW4 and BYCDW8

The antimicrobial activities of the compounds isolated from the strain BYCDW4 and BYCDW8 is shown in Table 3. Under the test concentration of 30 μg/filter paper, compound **1** [5-hydroxyramulosin **(1a)**:biatriosporin M **(1b)** = 2:1] exhibited some inhibitory activities against all the test pathogens. More specifically, the inhibitory effect on *E. coli* was the most significant with the IZD of 13.7 mm, which was slightly weaker than that of positive gentamicin sulfate with IZD of 17.7 mm. Compound **1** also showed good antimicrobial activities against *C. albicans*, *B. subtilis*, and *S. aureus* with the IZD of 14.33, 12.17, and 11.33 mm, respectively, which were comparable with those of the positive control. Compound **2** exhibited medium inhibitory activity against *B. subtilis* and *S. aureus*, with the IZD range of 8.32–9.13 mm, while it had no inhibition effect on *C. albicans*.

**TABLE 3:** Antimicrobial activities of compounds isolated from BYCDW4 and BYCDW8 against the test pathogens (mm).

Compounds	*E. coli*	*C. albicans*	*B. subtilis*	*S. aureus*
1	13.67 ± 1.15	14.33 ± 2.57	12.17 ± 0.76	11.33 ± 0.58
2	6.53 ± 0.30	NI	8.32 ± 0.07	9.13 ± 0.17
PC^a^	17.67 ± 0.29	19.17 ± 0.76	19.17 ± 0.29	21.33 ± 0.58

*^*a*^PC as the positive control, gentamicin sulfate and amphotericin as the positive control of pathogenic bacteria and yeast, respectively; results are presented as the mean ± standard; “NI” means not inhibited; the concentration for the test is 30 μg/filter paper.*

## Discussion

Microorganisms isolated from the bioprospecting underexploited environments with rich microbial biodiversity has been an important source of active natural products and new microorganism resources. A great variety of cultivable microbial flora was reported to exist in insects (Douglas, 2015; Shao et al., 2017; Blackwell and Vega, 2018). These important microbial resources were increasingly reported to be important sources of new natural products (Carr et al., 2012; Adnani et al., 2017; Xiao et al., 2017). In this study, the diversity of symbiotic fungi associated with *O. formosanus* and endophytic bacteria were studied. Thirty-nine fungi, including some potential new species, were isolated by culture-dependent method and molecular biological identification. To our knowledge, this is the first time that the phylogenetic diversity of cultivable and biological activity screening of secondary metabolites of fungi isolated from *O. formosanus* was studied, which will provide an important microbial resource for the discovery of new species and natural products.

Today, multidrug resistance of pathogens represents one of the major challenges to treat infectious diseases in community- and hospital-acquired infection diseases. There is also an urgent need for new antibiotics against gram-negative bacteria which have already been resistant to existing carbapenems and third-generation cephalosporins (Luther et al., 2019). In our study, the crude extract of potential new fungus BYCDW4 showed especially notable inhibitory effect against gram-negative *E. coli* (ATCC 8739) with the IZD of 21.07 mm, which was stronger than that of the positive gentamicin sulfate. The inhibition effect of crude extract against *E. coli* was better than purified compound **1** with the IZD of 13.6 mm. This might be the result of the synergistic action of multiple compounds, or the more active compounds have not been separated from the crude extract. Further research on other metabolites of BYCDW4 will be expected to develop new antibiotics for the treatment of gram-negative bacteria.

The potential new isolates BYCDW24 and BYCDW25 (in the order Pleurotheciales) presented selectively antimicrobial activities against the pathogens. BYCDW25 had good inhibitory activity against *C. albicans* and nearly no inhibition to *E. coli*, while BYCDW24 showed certain inhibitory effect on *E. coli* but no effect on *C. albicans*. As we reported, the BYCDW24 and BYCDW25 shared the same evolutionary relationship except the BYCDW25 harboring one endophytic bacterium. Similar to endobacteria of insects, the endobacteria of fungi showed a range of behaviors from mutualism to antagonism (Bonfante and Desiro, 2017). It has been reported that endobacterium affects the activity of host fungus (Partida-Martinez and Hertweck, 2005; Lackner and Hertweck, 2011). Whether the endobacterium from BYCDW25 played an important role in the biological activity should be further explored.

It is reported that the majority of symbiotic bacteria was identified as *Bacillus* from the fungus-growing termite (Mathew et al., 2012; Zhu et al., 2012). In our experiments, 66.7% (6/9) symbiotic bacteria from the cultivable fungi were identified as *Bacillus*, which proved that these dominant bacterial symbionts can also inhabit fungi isolated from the gut of *O. formosanus*.

## Conclusion

In this study, 39 fungi were isolated and identified from *O. formosanus*, which were belonging to two phyla and four classes. Furthermore, nine bacterial 16S rRNA sequences were obtained from total fungal genomic DNA. All bacterial symbionts belonged to four genera: *Bacillus*, *Methylobacterium*, *Paenibacillus*, and *Trabulsiella.* The antimicrobial bioassay showed that 25 fungal extracts (64%) exhibited antibacterial activities against at least one of the tested bacterial strains. Furthermore, the secondary metabolites **1** [5-hydroxyramulosin **(1a)**:biatriosporin M **(1b)** = 2:1] from the *Pleosporales* sp. BYCDW4 exhibited potent antimicrobial activities against all tested pathogens with the IZD of more than 11.3 mm, which were comparable with those of the positive control. 1-(2,5-Dihydroxyphenyl)-3-hydroxybutan-1-one (**2**) from the *Microdiplodia* sp. BYCDW8 showed medium antibacterial activities against *B. subtilis* and *S. aureus*. In conclusion, the report showed the diversity of insect symbionts had a potential to develop the resource of new species and antibiotics.

## Data Availability Statement

The raw data supporting the conclusions of this article will be made available by the authors, without undue reservation, to any qualified researcher.

## Author Contributions

YZ designed the research and supervised the study. XX, MS, CY, XY, FS, and ZM performed the experiments and analyzed the data. XX and MS wrote the manuscript. All authors revised the manuscript and approved the final version for submission.

## Conflict of Interest

The authors declare that the research was conducted in the absence of any commercial or financial relationships that could be construed as a potential conflict of interest.
