# Supplementary material for: Diversity, Bacterial Symbionts, and Antimicrobial Potential of Termite-Associated Fungi
Source: Front Microbiol. 2020 Mar 13;11:300. doi: 10.3389/fmicb.2020.00300 (PMC7082625; doi:10.3389/fmicb.2020.00300)
Supplement: Supplementary file 1 [file Data_Sheet_1.docx]

**Supplementary Materials:**

**Diversity, Bacterial Symbionts and Antimicrobial Potential of Termite-Associated Fungi**

*Xiao Xu^1, 3,^*^†^*, Mingwei Shao^1, 3,^*^†^*, Caiping Yin^1^, Zhenchuan Mao^2*^, Jingjing Shi^3^, Xinyuan Yu^3^, Ying Wang^3^, Feifei Sun^3^* *and Yinglao Zhang^1, 3 *^*

*^1^ School of Life Sciences, Anhui Agricultural University, Hefei, 230036, PR China ^2^Institute of Vegetables and Flowers, Chinese Academy of Agricultural Sciences, Beijing 100081, China*

*^3^ College of Chemistry and Life Science, Zhejiang Normal University, Jinhua, 321004, PR China*

***Correspondence:

E-mail address:

maozhenchuan@caas.cn (Z.C. Mao), Tel.: +86-010-82109545;

zhangyl@ahau.edu.cn (Y. L. Zhang), Tel.: +86-551-6578-6129

†These authors have contributed equally to this work.

**Table S1**. The isolation media

| HV | [Humic](C:/Users/%E5%BE%90%E6%99%93/AppData/Dict/8.5.1.0/resultui/html/index.html#/javascript:;) [acid](C:/Users/%E5%BE%90%E6%99%93/AppData/Dict/8.5.1.0/resultui/html/index.html#/javascript:;) 1.0 g, KC11.0 g, Na_2_HPO_4_ 0.5 g, CaC0_3_ 0.02 g, MgSO_4_•7H_2_O 0.5 g, FeSO_4_•7H_2_O0.01g, agar15.0 g, HV Multi-Vitamins (HV Multi-Vitamins: thiamine 0.05 g,riboflavin 0.05g, inose 0.05 g,[pantothenic](C:/Users/%E5%BE%90%E6%99%93/AppData/Dict/8.5.1.0/resultui/html/index.html#/javascript:;) [acid](C:/Users/%E5%BE%90%E6%99%93/AppData/Dict/8.5.1.0/resultui/html/index.html#/javascript:;) 0.05 g, ofp-aminobenzoic acid 0.05g, [vitamin](C:/Users/%E5%BE%90%E6%99%93/AppData/Dict/8.5.1.0/resultui/html/index.html#/javascript:;) [B_6_](C:/Users/%E5%BE%90%E6%99%93/AppData/Dict/8.5.1.0/resultui/html/index.html#/javascript:;) 0.05g, biotin 0.025 g, niacin 0.05 g, ddH_2_O 100 mL)l.0 mL, H_2_O 1000 mL, pH 7.2-7.4. |
| --- | --- |
| ISP 2 | Yeast extract 4.0 g, glucose 4.0g, malt extract 10.0 g, Microsaline solution 1 mL, ddH_2_O 1000 mL, pH 7.2. |
| ISP 3 | Oatmeal 20.0g, Microsaline solution mL, ddH_2_O1000 mL, pH 7.2. |
| M7 | Sodium propionate 2.0 g, KNO_3_ 0.1g, glycine betaine 1.25 g, MgSO_4_•7H_2_O 0.05 g, K_2_HPO_4_•3H_2_0 0.2 g, CaCO_3_ 0.2 g, FeSO_4_•7H_2_O 0.01 g, NaCl 10.0 g, ddH_2_O 1000 mL, pH7.2. |
| YIM-2 | Inulin 2.0 g, cystine 0.5 g,K_2_HPO_4_ 1.0 g,NaCIO 0.8 g, MgSO_4_ 0.5 g,CaCO_3_ 0.l g, thiamine 0.5 mg, riboflavin 0.5 mg, pyridoxine 0.5 mg, biotin 0.5 mg,niacin 0.5 mg, Microsaline solution 1 mL, ddH_2_O 1000 mL. |
| Modified HV | Starch 2.0 g, KNO_3_ 0.5 g,KCl 1.7 g, MgSO_4_ 0.5 g, Na_2_HPO_4_ 0.5 g,CaCO_3_ 0.02 g,FeS0_4_ 0.01 g, Microsaline solution 1 mL, ddH_2_O 1000 mL, pH7.2. |
| Trehalose-proline | Trehalose 5.0 g, proline 1.0 g,(NH_4_)_2_SO_4_ l.0g, NaCl l.0 g,CaCl_2_ 2.0 g, K_2_HPO_4_ l.0 g,MgSO_4_•7H_2_O 1.0 g, Microsaline solution l ml, H_2_O 1000 mL, pH7.2. |
| YIM-3 | Mannose4.0 g, histidine2.0g,K2HPO_4_ l.0 g, NaCl 0.8g, MgSO_4_ 0.5 g,CaCO_3_ 0.1 g, thiamine 0.5 g, riboflavin 0.5 g, pyridoxine 0.5 g, biotin 0.5 mg, niacin 0.5 mg, Microsaline solution l mL, ddH_2_O 1000 mL, pH7.2. |
| Modified proline | Proline 5.0 g, ddH_2_O 1000 mL, PH7.2. |
| Modified Gauze's synthetic medium No. 2 | Glucose l.0 g, peptone 0.5 g, tryptone 0.3 g, NaCl 0.5 g, microsaline solution lmL, ddH_2_O 1000 mL, pH7.2 |

**Table S2**

The taxonomic classification of bacterial symbionts from cultivable fungi associated with *O. formosanusis.*

| **Isolate code** | **Closest**  **match** | **Accession**  **no.** | **Proposed**  **identity** | **Coverage/Max ident** | **GenBank**  **no.** |
| --- | --- | --- | --- | --- | --- |
| BYCDW1a | *Bacillus subtilis* | EF541143 | *Bacillus subtilis* | 99/99 | MG825089 |
| BYCDW4a | *Bacillus velezensis* | MF620083 | *Bacillus velezensis* | 99/99 | MG825090 |
| BYCDW6a | *Bacillus megaterium* | MG008657 | *Bacillus megaterium* | 99/99 | MG825091 |
| BYCDW13a | *Methylobacterium tardum* | KR085941 | *Methylobacterium tardum* | 98/99 | MG825092 |
| BYCDW19a | *Bacillus tequilensis* | KC172053 | *Bacillus tequilensis* | 99/99 | MG825093 |
| BYCDW20a | *Bacillus subtilis* | KF001839 | *Bacillus subtilis* | 99/99 | MG825094 |
| BYCDW25a | *Paenibacillus naphthalenovorans* | NR_028817 | *Paenibacillus naphthalenovorans* | 99/98 | MG825095 |
| BYCDW29a | *Trabulsiella guamensis* | KC434962 | *Trabulsiella guamensis* | 98/98 | MG825096 |
| BYSTW3a | *Bacillus subtilis* | KX869961 | *Bacillus subtilis* | 99/99 | MG825097 |

| **Table S3**  Crystal data and structure refinement for **1a**. | |
| --- | --- |
| Identification code | **1a** |
| Empirical formula | C_10_H_14_0_4_ |
| Formula weight | 198.09 |
| Temperature/K | 296.15 |
| Crystal system | orthorhombic |
| Space group | P2_1_2_1_2_1_ |
| a/Å | 4.7270(3) |
| b/Å | 14.0434(9) |
| c/Å | 14.4282(9) |
| α/° | 90 |
| β/° | 90 |
| γ/° | 90 |
| Volume/Å^3^ | 957.79(11) |
| Z | 28 |
| ρ_calc_g/cm^3^ | 1.409 |
| μ/mm^‑1^ | 0.130 |
| F(000) | 420.0 |
| Crystal size/mm^3^ | 0.32 × 0.28 × 0.11 |
| Radiation | MoKα (λ = 0.71073) |
| 2Θ range for data collection/° | 4.048 to 55.22 |
| Index ranges | -6 ≤ h ≤ 6, -18 ≤ k ≤ 18, -18 ≤ l ≤ 18 |
| Reflections collected | 31984 |
| Independent reflections | 2209 [R_int_ = 0.0683, R_sigma_ = 0.0227] |
| Data/restraints/parameters | 2209/0/127 |
| Goodness-of-fit on F^2^ | 1.146 |
| Final R indexes [I>=2σ (I)] | R_1_ = 0.0419, wR_2_ = 0.1105 |
| Final R indexes [all data] | R_1_ = 0.0481, wR_2_ = 0.1173 |
| Largest diff. peak/hole / e Å^-3^ | 0.28/-0.19 |
