# Supplementary material for: Diversity, Bacterial Symbionts, and Antimicrobial Potential of Termite-Associated Fungi
Source: Front Microbiol. 2020 Mar 13;11:300. doi: 10.3389/fmicb.2020.00300 (PMC7082625; doi:10.3389/fmicb.2020.00300)
Supplement: Supplementary file 2 [file Data_Sheet_2.pdf]

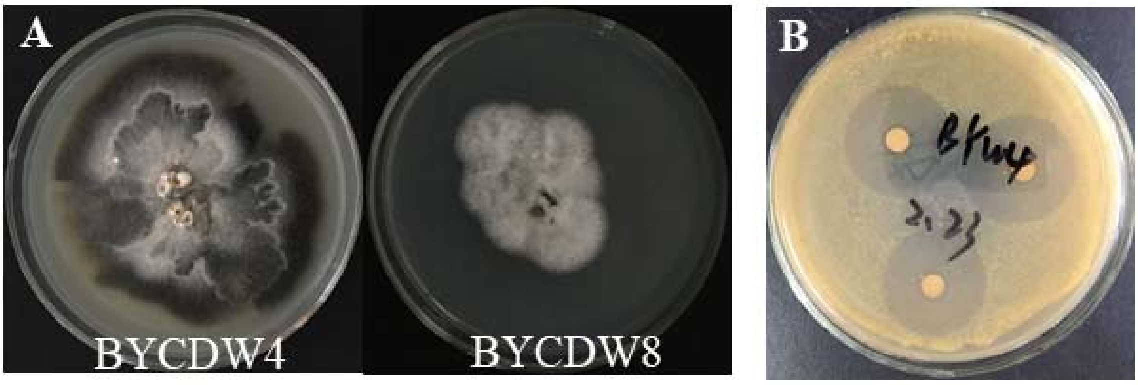


**Figure S1.** Colony of strains BYCDW4 and BYCDW8 (A), and antibacterial activity of BYCDW4 crude extract against *Staphylococcus aureus* (B)**.**

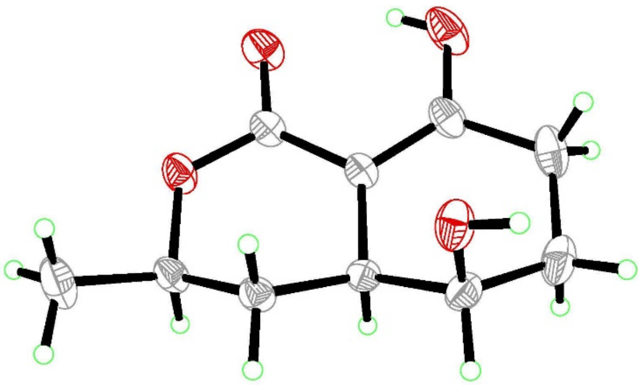

**Figure S2.** X-ray crystallographic **1a**.
